# Mapping the cardiovascular burden in transgender individuals: a systematic review and meta-analysis

**DOI:** 10.3389/fpubh.2026.1755343

**Published:** 2026-07-14

**Authors:** Mallicka Mallicka, Deep Shikha, Sai Chandan Das, Richa Sinha

**Affiliations:** 1 Department of Community Medicine, Integral University, Lucknow, India; 2 Department of Community Medicine, Himalayan Institute of Medical Sciences, Swami Rama Himalayan University, Dehradun, India; 3 Department of Community Medicine, Kalinga Institute of Industrial Technology Deemed to be University, Bhubaneswar, India; 4 Department of Community Medicine, Government Doon Medical College, Dehradun, India

**Keywords:** cardiovascular disease, transgender persons, gender-affirming hormone therapy, myocardial infarction, hypertension, stroke, systematic review, meta analysis

## Abstract

**Background:**

Transgender individuals represent a marginalised population, often exposed to unique psychosocial stressors, limited healthcare access, and social exclusion, all of which may contribute to an increased risk of cardiovascular disease (CVD). However, evidence focusing exclusively on the cardiovascular outcomes in this group remains limited. This systematic review and meta-analysis aimed to estimate the pooled prevalence of major cardiovascular morbidities among transgender adults.

**Methods:**

A systematic search of five databases (PubMed, PubMed Central, Embase, Scopus, and Google Scholar) was conducted to identify observational studies reporting cardiovascular outcomes in transgender individuals aged ≥18 years. The study protocol was registered with PROSPERO (CRD42022383213). The inclusion criteria were cross-sectional, cohort, and case-control studies published in English. Data extraction and quality assessment were independently performed by two reviewers using standardised tools. A meta-analysis was conducted using a random-effects model, and publication bias was assessed using funnel plots and Egger's test.

**Results:**

10 eligible studies that focused on transgender individuals were included in the final analysis. The pooled prevalence rates were hypertension 21% (95% CI: 12–36; *I*^2^ = 99.0%), stroke/CVA 5% (95% CI: 3–8; *I*^2^ = 84.3%), coronary artery disease (CAD) 3% (95% CI: 1–19; *I*^2^ = 98.8%), and myocardial infarction (MI) 7% (95% CI: 6–9; *I*^2^ = 75.5%). Funnel plots indicated a symmetrical distribution for hypertension and stroke, suggesting low publication bias, whereas asymmetry was noted for CAD and MI.

**Conclusion:**

This review revealed a substantial burden of cardiovascular morbidity among transgender individuals, warranting routine screening and targeted preventive strategies in this population. Policymakers and healthcare providers should consider inclusive approaches to reduce cardiovascular risk. Further longitudinal studies and comparative analyses of cisgender populations are essential for guiding evidence-based interventions.

**Systematic review registration:**

https://www.crd.york.ac.uk/prospero/display_record.php?ID=CRD42022383213, identifier: CRD42022383213.

## Introduction

Transgender individuals are those whose gender identities differ from the gender assigned to them at birth (1). Although global estimates of transgender populations remain imprecise, available literature suggests a prevalence of approximately 0.5%, equating to nearly 25 million individuals worldwide (2). Variability in these estimates may reflect differences in sociocultural acceptance, legal recognition, healthcare access, and reporting practices across regions. Increasing numbers of transgender individuals worldwide are seeking gender-affirming hormone therapy (GHT), highlighting the growing importance of understanding long-term cardiovascular health outcomes in this population. In India, approximately 4.88 lakh individuals identify as transgender, reflecting the broader global need for inclusive and gender-affirming healthcare services (3, 4).

Approximately 80% of transgender people in the United States reported using or planning to use gender affirming hormone therapy (GHT), according to the National Transgender Discrimination Survey Report on Health and Health Care. However, estimates may differ between nations and may not be generalized, as sociocultural context, gender affirming care, legal acknowledgement and healthcare access vary substantially (5) Nevertheless, with increasing global visibility and evolving socio-cultural acceptance of transgender individuals, there is an urgent need for healthcare system and providers across both public and private sectors to address their specific medical needs (6). GHT aims to align secondary sex characteristics with one's gender identity, for example, administering testosterone in transgender men to achieve male physiological levels and estrogen combined with anti-androgens such as gonadotropin-releasing hormone analogues or cyproterone acetate in transgender women (7, 8).

Recent research suggests that transgender individuals may experience a high burden of CVD risk factors and an elevated risk of adverse cardiovascular outcomes (9). Data from the self-reported Behavioural Risk Factor Surveillance System (BRFSS) survey indicates that transgender men have over twice the prevalence of myocardial infarction compared to cisgender men, and more than four times compared to cisgender women and an increased prevalence among transgender women compared to cisgender women, although the difference was not statistically significant compared to cisgender men. As the findings were specific to the United States and were based on self-reported diagnoses, these should be interpreted accordingly and may not reflect the observations among other populations (10, 11).

However, the study of cardiovascular outcomes in transgender populations presents several challenges. The interpretation of cardiovascular outcomes in transgender populations is challenging because many study cohorts are relatively young, whereas cardiovascular events typically manifest later in life (11).

This age mismatch, combined with the relatively low number of cardiovascular events and limited sample sizes in individual studies, geographical concentration, and limited representation from low- and middle-income countries, restricts the statistical power to detect meaningful associations (12).

Currently, there is a significant paucity of robust evidence focusing solely on cardiovascular disease (CVD) outcomes in transgender individuals. Furthermore, most extant studies examining cardiovascular outcomes in transgender populations are concentrated in specific regions, predominantly high-income Western countries such as the United States and the Netherlands, with limited representation from low- and middle-income settings.

Although numerous observational studies have examined cardiovascular risk factors in transgender populations, the current evidence base is fragmented, geographically constrained, and characterized by methodological heterogeneity. Few studies have synthesized pooled prevalence estimates focused exclusively on major cardiovascular morbidities within this population. This systematic review and meta-analysis aim to address this significant evidence gap by providing consolidated, prevalence estimates for key cardiovascular diseases among transgender individuals, utilizing data from diverse global contexts to improve generalizability.

## Research question

What is the prevalence of cardiovascular morbidities in transgender populations?

### Protocol registration

This systematic review and meta-analysis were conducted in accordance with the preferred reporting items for systematic reviews and meta-analyses (PRISMA) 2020 guidelines. The study protocol was prospectively registered in the PROSPERO international database (registration number CRD42022383213), ensuring transparency and minimising duplication.

PEO Statement (participants, exposure, outcome)

- P (Participants): transgenders aged 18 years and above
- E (Exposure): being transgender (including transgender men, transgender women)
- O (Outcome): prevalence of cardiovascular morbidities, including hypertension, myocardial infarction (MI), stroke/cerebrovascular accident (CVA), and coronary artery disease (CAD)

### Inclusion citeria

- Observational studies (cross-sectional, case-control, retrospective, prospective cohort, and national survey-based studies)
- Grey literature, including unpublished data and preprints with methods and results sections
- Studies that reported cardiovascular outcomes specifically among transgender individuals
- Studies with a minimum sample size of 30 transgender participants were included to reduce small-study effects and improve the statistical stability of pooled prevalence estimates
- Articles published in English

### Exclusion criteria:

- Literature review, meta-analyses, randomised controlled trials, editorials, commentaries, and case reports
- Duplicate publications
- Studies involving cisgender populations or where transgender-specific data could not be extracted
- Studies that do not report cardiovascular outcomes as primary or secondary endpoints.
- Articles published in languages other than English

### Search strategy

**Figure 1:**
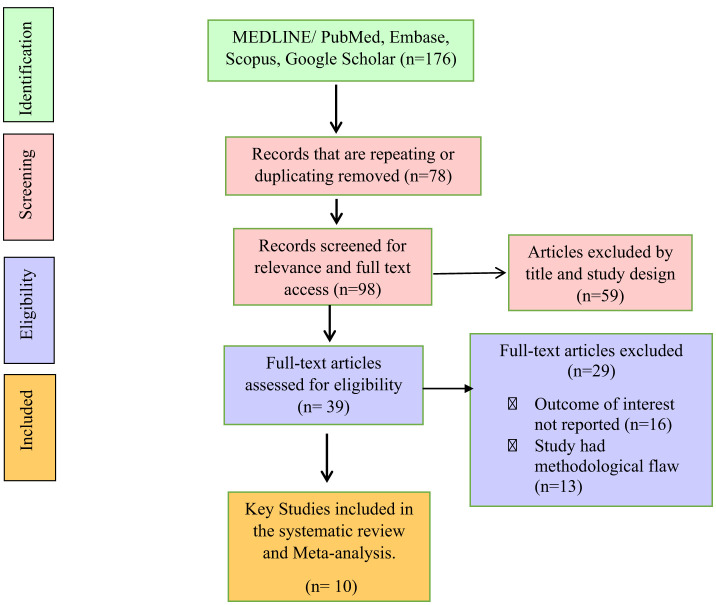
PRISMA flowchart detailing results of literature search and study screening of research studies.

A comprehensive and sensitive search strategy was developed in consultation with a medical librarian to capture all relevant literature. Five databases were searched: PubMed, PubMed Central (PMC), Embase, Scopus, and Google Scholar, covering literature from database inception until December 31, 2023, representing the most recent complete calendar year at the time of protocol finalization. The search terms used were a combination of MeSH headings and keywords prioritizing broad cardiovascular outcome terms, including: “Transgenders,” “transgender population,” “cardiovascular risk,” “hypertension,” “myocardial infarction,” “stroke,” and “cardiovascular risk factors”to maximize sensitivity and capture heterogenous reporting across databases. Boolean operators (AND, OR) were applied to optimise the search sensitivity, example: (‘transgender persons' OR ‘transgender population') AND (‘cardiovascular disease' OR ‘hypertension' OR ‘stroke' OR ‘myocardial infarction' OR ‘coronary artery disease'). Handsearching of reference lists of included articles and grey literature was searched through structured screening of preprint servers and institutional repositories, including medRxiv and WHO IRIS, using predefined keyword combinations consistent with the primary database search strategy to ensure completeness. The study selection flow is presented in Figure 1.

### Selection of studies

All citations were exported to EndNote X8 for de-duplication. Two independent reviewers (reviewers A and B) screened the titles and abstracts of the retrieved articles. Full-text articles were assessed for eligibility by using predefined inclusion criteria. In cases of ambiguity or disagreement, resolution was achieved through discussion and, if needed, a third senior reviewer (Reviewer C) provided arbitration. All the reasons for exclusion in the full-text screening stage were documented. The PRISMA 2020 flow diagram (Figure 1) summarizes the study selection process, including identification of records through database searching, removal of duplicate records, title and abstract screening, full-text eligibility assessment, reasons for exclusion, and final inclusion of studies in the systematic review and meta-analysis

### Data extraction

Two reviewers independently extracted data from the eligible studies using a pre-piloted, standardised extraction form. The extracted variables included author name, year of publication, country/region, study design, sample size, population characteristics (age and sex identity subgroups), type and duration of hormone therapy (if applicable), cardiovascular outcome(s) assessed, outcome definitions, and prevalence or event rates with corresponding confidence intervals. If data were missing or unclear, the authors were contacted via email. Data were cross-verified, and discrepancies were resolved by consensus or third-party adjudication. Where cardiovascular data were disaggregated by transgender subgroups (e.g., trans-men vs. trans-women), they were treated as distinct datasets.

#### Assessment of methodological quality

**Figure 2:**
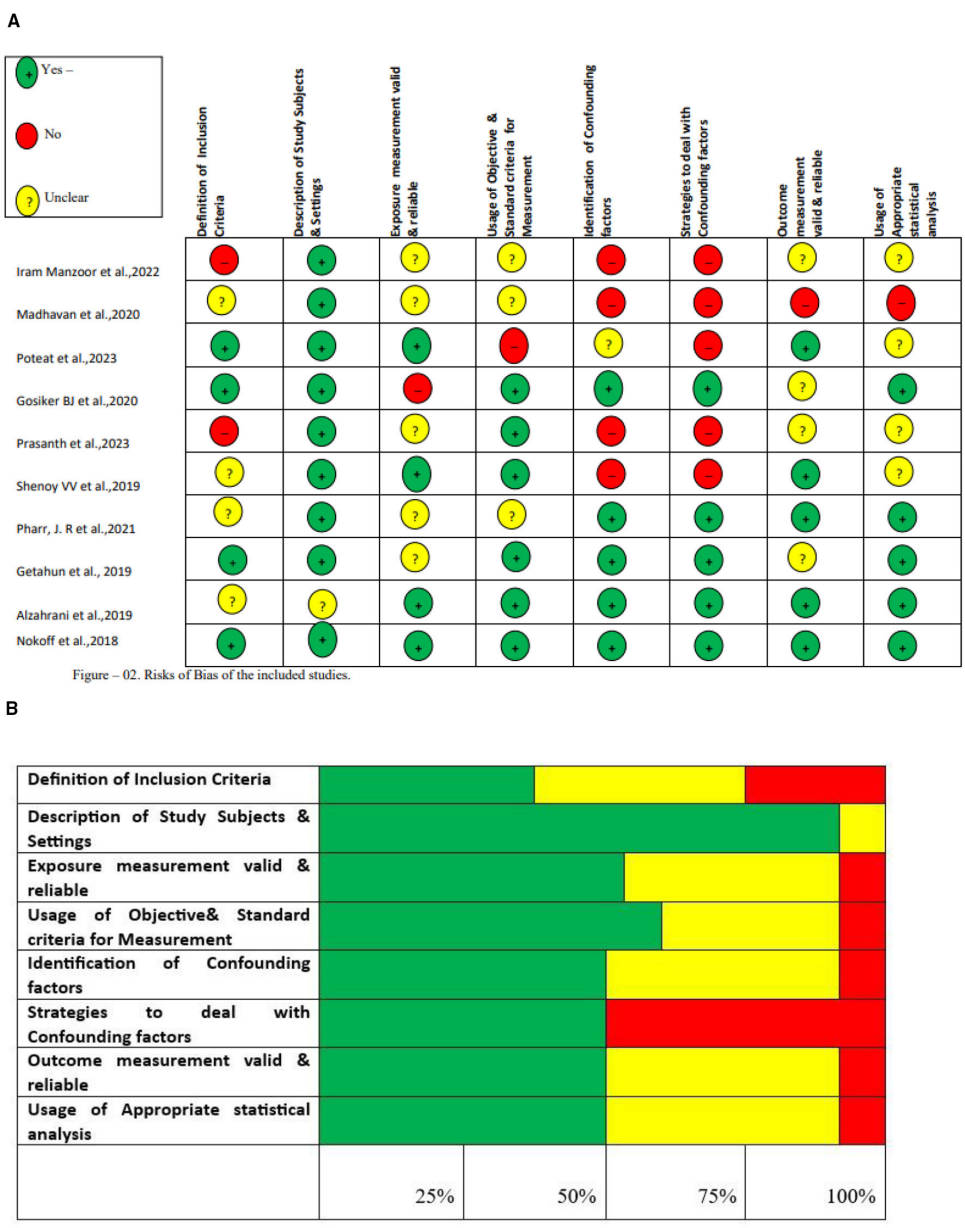
Assessment of methodological quality using the Joanna Briggs Institute (JBI) critical appraisal tool: **(A)** risk of bias summary and **(B)** risk of bias graph.

The risk of bias in the included studies was assessed independently by two reviewers using the Joanna Briggs Institute (JBI) critical appraisal tool appropriate for each study design. The criteria evaluated included the sampling strategy, measurement of exposure and outcome, adjustment for confounders, and clarity of statistical analysis. Each study was rated as having low, moderate, or high risk of bias. Two studies (Getahun et al. and Alzahrani et al.) reported cardiovascular outcomes separately for transgender men and transgender women and were therefore treated as independent datasets in the meta-analysis to preserve subgroup-specific prevalence estimates. Of the 10 studies with 12 datasets analysed, four studies were rated as low risk, three moderate risk, and three high risk of bias. The domain-wise methodological quality assessment of included studies using the JBI critical appraisal tool is summarized in Figure 2 and discussed narratively within the manuscript.

### Data synthesis and analysis

Meta-analysis was performed in R (version 4.3.0) using the meta and metafor packages. Due to the expected clinical and methodological heterogeneity, a random-effects model (DerSimonian and Laird method) was used for all pooled estimates. Although the DerSimonian and Laird estimator is widely used in prevalence meta-analyses, it may underestimate variance in the presence of substantial heterogeneity; alternative estimators such as restricted maximum likelihood (REML) may provide more robust variance estimates. The pooled prevalence rates were calculated separately for each cardiovascular outcome (hypertension, MI, stroke, CAD). Forest plots were generated to visualise individual and pooled prevalence estimates, and *I*^2^ statistics were calculated to assess heterogeneity; *I*^2^ values >75% were considered substantial. Subgroup analyses were performed when data were available, for example, by gender identity (transgender men vs. transgender women), geographic region, and hormone therapy use. Sensitivity analyses were conducted by sequential exclusion of studies categorized as having high risk of bias to evaluate the robustness and stability of pooled prevalence estimates across cardiovascular outcome. Funnel plots and Egger's regression test were used to assess publication bias, with *p* < 0.05 considered significant. All analyses were two-tailed and were reported with 95% confidence intervals.

## Results

Table 1 presents data from 10 studies conducted across India, the USA, Pakistan, including a retrospective study conducted in the U.S. states of Georgia and California, focusing on the prevalence of cardiovascular comorbidities among transgender individuals, particularly hypertension (HTN), cerebrovascular accident (CVA), coronary artery disease (CAD), and myocardial infarction (MI). Sample sizes varied widely, ranging from 54 to over 340,000 participants. The prevalence of hypertension ranges from 1.4% (Manzoor I, 2022, Pakistan) (14) to as high as 73.0% (Gosikar, , USA) (15), reflecting considerable heterogeneity possibly due to differences in geography, population subgroups (transgender men vs. women), and data collection methods. Similarly, the MI rates ranged from 4.4 to 7.9%, CVA rates ranged from 2.3 to 7.3%, and CAD rates ranged from 1.7 to 13.9%. Most studies used either face-to-face interviews or retrospective data from national surveys, contributing to both breadth and variability in the findings (Table 1).

**Table 1:** Characteristics of the study included in the systematic review on prevalence of cardiovascular diseases among the transgender population.

References	Country	Study design and type of data collection	Study participants	Sample size	Outcome
Prasanth BK, (27)	India	Descriptive cross-sectional study Face to face interview	TG > 18 years	145	HTN = 48.50% CVA/stroke = 7.30% CAD/ANGINA/CHD = 13.90%
Shenoy VV, (31)	India	Descriptive cross-sectional study Face to face interview	Transwomen	54	HTN = 11.10% CVA/stroke = 3.70%
Pharr JR, (16)	USA	Cross-sectional, telephonic survey	TG > 18 years	2,827	HTN = 31.30% CVA/stroke = 6.50% CAD/ANGINA/CHD = 7.10% MI = 7.90%
Alzahrani T, (1, 2, 10)	USA	Retrospective study, data obtained from the behavioural risk factor surveillance system data from 2014 to 2017, USA. From records	Transgender men	1,267	HTN = 26.00% MI = 7.20% HTN = 34.80% MI = 7.80%
			Transgender women	340,365	
Madhavan M, (25)	India	Descriptive cross-sectional study Face to face interview	Transgender men and women	200	**HTN** **=** 6.50%
Poteat TC, (32)	USA	Cross-sectional study as baseline data was taken from the LITE plus study. From records	Transgender men and women	102	**HTN** **=** 23.00%
Getahun D, (12, 13)	Georgia and California	Retrospective cohort study, from the records	Transgender men	2,118	**HTN** **=** 9.30% **CAD/ANGINA/CHD** **=** 1.70% **HTN** **=** 16.00% **CAD/ANGINA/CHD** **=** 1.90%
			Transgender women	2,842	
Gosikar BJ, (15)	USA	Cross-sectional study, Face to face interview	Transwomen	221	**HTN** **=** 73.00%
Manzoor I, (14)	Pakistan	Analytical cross-sectional study, Face to face interview	Transgender men and women	214	**HTN** **=** 1.40%
Nokoff NJ, (17)	USA	Retrospective study, Data retrieved from the 2015 behavioural risk factor sance system survey. From the records	Transgender men and women	764	**HTN** **=** 27.90% **CVA/stroke** **=** 2.30% **CAD/ANGINA/CHD** **=** 3.20% MI = 4.40%

TG, transgender; HTN, hypertension; CVA, cerebrovascular accident; CAD, coronary artery disease and MI, myocardial Infarction.

Study-specific confidence intervals and weighting contributions are presented in the corresponding forest plot.

**Figure 3:**
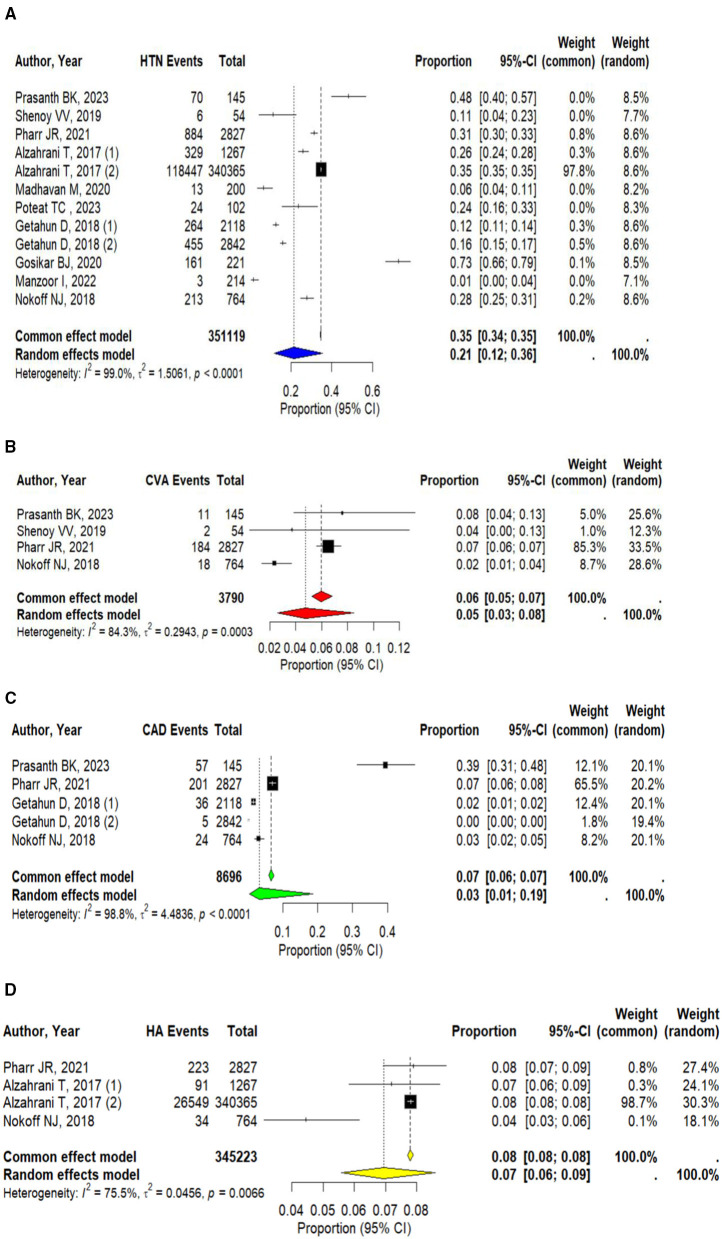
**(A)** Forest plot for pooled prevalence of HTN among the transgenders **(B)** Forest plot for pooled prevalence of CVA among the transgenders **(C)** Forest plot for pooled prevalence of CAD among the transgenders **(D)** Forest plot for pooled prevalence of Myocardial Infarction mong the transgenders.

This forest plot summarises a meta-analysis of selected studies that assessed the proportion of hypertension (HTN) events. The reported proportions vary widely, ranging from 0.01 to 0.73, reflecting substantial between-study variability. The pooled estimate using the fixed-effect model was 0.35 [95% CI: 0.34–0.35], heavily influenced by the largest study (Alzahrani T, 2019 (10)), which contributed 97.8% of the weight. However, due to very high heterogeneity (*I*^2^ = 99.0%, *p* < 0.0001), the random-effects model is more appropriate, yielding a more conservative and uncertain pooled estimate of 0.21 [95% CI: 0.12–0.36]. This high heterogeneity suggests major differences in the populations, methodologies, or definitions of hypertension across studies. Overall, although hypertension is prevalent in these populations, the actual proportion is likely closer to 21%, with notable variability across settings (Figure 3A).

This forest plot shows a meta-analysis of four studies assessing the proportion of cerebrovascular accident (CVA) events. The proportions reported in individual studies range from 0.02 to 0.08, with the pooled estimate under the fixed-effects model being 0.06 [95% CI: 0.05–0.07], and under the random-effects model, 0.05 [95% CI: 0.03–0.08]. There was significant heterogeneity among the studies (*I*^2^ = 84.3%, *p* = 0.0003), indicating variability beyond chance, which justifies the use of a random-effects model. The wide confidence intervals in some studies and the variation in weights show that while one study (Pharr JR, 2021) dominates the fixed-effects analysis, the random-effects model balances contributions more evenly (16). Overall, the pooled estimate suggests that approximately 5% of the studied population experienced CVA events, with moderate heterogeneity-related uncertainty (Figure 3B).

This forest plot presents a meta-analysis of five studies that reported the proportion of CAD (coronary artery disease) events. The individual study estimates ranged widely from 0 to 39%, with significant heterogeneity (*I*^2^ = 98.8%, *p* < 0.0001), suggesting substantial variability across studies. Under the fixed-effect model, the pooled proportion was 0.07 [95% CI: 0.06–0.07], whereas the random-effects model, which accounts for heterogeneity, yielded a lower, more uncertain estimate of 0.03 [95% CI: 0.01–0.19]. The large confidence interval and high heterogeneity suggest that the true proportion of CAD events may vary considerably across study populations and methods, making the random-effects estimate more appropriate for interpretation (Figure 3C).

This forest plot presents a meta-analysis of four studies assessing the proportion of myocardial infarction (MI) events. The reported proportions across studies range from 0.04 to 0.08. The pooled estimate under the fixed-effect model was 0.08 [95% CI: 0.08–0.08], while the random-effects model, which accounts for between-study differences, yielded a slightly lower pooled proportion of 0.07 [95% CI: 0.06–0.09]. There was a statistically significant moderate heterogeneity among the studies (*I*^2^ = 75.5%, *p* = 0.0066), suggesting that the studies were not completely homogeneous. One very large study (Alzahrani T, [2] (10)) dominated the fixed-effects analysis (98.7% of the weight), but the random-effects model mitigates this influence. Overall, the meta-analysis suggests that approximately 7% of individuals across these studies experienced myocardial infarction, with some variability across the study populations (Figure 3D).

### Publication bias

**Figure 4:**
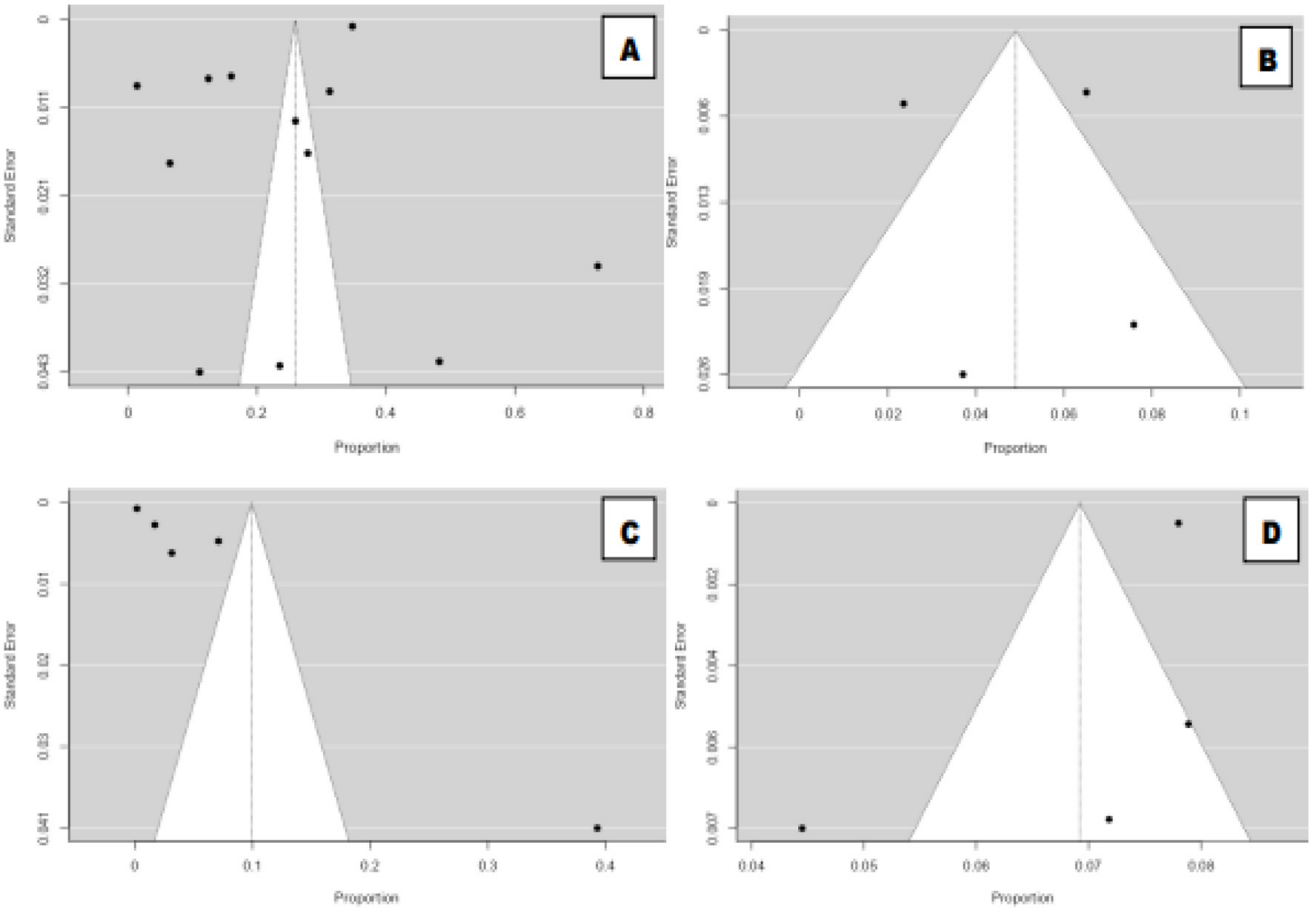
Funnel plots for publication bias **(A)**: HTN, **(B)**: CVA/STROKE, **(C)**: CAD, **(D)**: MI

Publication bias was assessed for all cardiovascular outcomes included in the meta-analysis by using funnel plot visualisation. Hypertension, myocardial infarction (MI), stroke/cerebrovascular accidents (CVA), and coronary artery disease (CAD) funnel plots were generated for each outcome to evaluate the symmetry of the study distribution. A symmetrical funnel plot suggests a low risk of publication bias, whereas asymmetry may indicate bias from selective reporting or small-study effects. Funnel plots for hypertension and stroke/CVA showed a relatively symmetrical distribution of studies, suggesting no significant publication bias for these outcomes. In contrast, funnel plots for CAD and MI showed marked asymmetry, raising concerns about publication bias in studies reporting these outcomes. These findings are illustrated in Figures 4A–D.

## Discussion

The transgender population constitutes a marginalised and underserved group that faces numerous social, psychological, and structural barriers to accessing healthcare. This group demonstrated poor health-seeking behaviour, primarily due to societal stigma and discrimination associated with their gender identity (13). These barriers not only limit their access to preventive and routine care but also increase their vulnerability to various health conditions, including cardiovascular diseases (CVDs). Gender-affirming hormone therapy (GAHT) and chronic psychosocial stress are thought to affect cardiovascular risk profiles in transgender people, but direct causal links to cardiovascular disease are not yet fully confirmed (14, 15, 17).

Despite these concerns, available literature on cardiovascular morbidity in transgender individuals remains limited. The current systematic review and meta-analysis address this knowledge gap by synthesising.

This review involved an extensive search of five major databases to identify studies reporting cardiovascular outcomes in transgender individuals. A total of ten studies were included in the final analysis. Transgender and gender-diverse (TGD) individuals often face stigma, discrimination, fear of disclosure, lack of legal documentation, and mistrust of healthcare and research institutions. These factors limit participation in population-based surveys and longitudinal cohorts, resulting in sparse and fragmented data. Many individuals who identify as transgender experience gender dysphoria and often seek GHT to suppress the development of their natal secondary sexual characteristics, while promoting those aligned with their affirmed gender. GHT has been associated with changes in cardiometabolic risk markers in some observational studies; however, causal links with cardiovascular events remain unestablished (18, 19). In addition to biological factors, multiple studies have highlighted the role of social determinants such as unemployment, lack of education, social exclusion, and low socioeconomic status as contributors to chronic psychological stress and adverse cardiovascular outcomes. Persistent poverty, housing instability, and engagement in informal, unstable, or marginal employment characterised by job insecurity, lack of legal protection, poor working conditions, and limited access to healthcare benefits (e.g., daily wage labour, sex work, undocumented work) further amplify health risks among transgender individuals (20–22).

The findings of this systematic review indicate that the pooled prevalence of hypertension, stroke/CVA, coronary artery disease (CAD), and myocardial infarction (MI) is notably high in transgender populations, suggesting that the CVD burden in this group is significant and should not be overlooked. Previous reviews have reported limited data on cardiovascular events among transgender individuals, although available evidence suggests a higher incidence in transgender women than in transgender men (23, 24). Our results reinforce the need for routine assessment of cardiovascular risk factors and addressing barriers such as healthcare stigma or limited access to the health sector. *Inclusive cardiovascular risk-screening strategies*, such as integration of blood pressure, lipid, and diabetes screening into gender-affirming care settings without assuming causality, are the need of the hour. In our meta-analysis, the pooled prevalence of hypertension among transgender individuals was found to be 26%. This is considerably higher than the prevalence reported in two cross-sectional studies by Madhavan et al. (16%) and Sivakami et al. (15%) (25, 26). These findings underscore the importance of blood pressure monitoring and early hypertension screening in transgender populations, particularly in those undergoing hormone therapy. This elevated prevalence may reflect the combined effects of psychosocial stress, extrapolated vascular effects of exogenous sex steroids described in non-transgender populations, and limited access to primary healthcare services. This represents diverse healthcare systems, hormone-access pathways, and social contexts. We now caution that: pooled estimates reflect context-specific risk environments, and findings may not be generalizable to regions with universal gender-affirming care or robust primary care access. The overall pooled prevalence of stroke/CVA among transgender individuals in this review was 5%, which is consistent with the 6.5% prevalence reported by Pharr et al. in a cross-sectional study (16). The increased risk of cerebrovascular events may be partly attributed to estrogen-based hormone therapy, especially when used at high doses or without regular medical supervision. These findings suggest the need for regular neurological evaluations and emphasise the importance of patient education regarding the safe and judicious use of hormonal therapies.

The pooled prevalence of CAD was 10%, which is comparable to the 13.9% prevalence reported by Prasanth et al. (27). This similarity reinforces the idea that transgender individuals face a comparable or potentially higher risk of CAD, likely influenced by both hormone therapy and behavioural factors, such as higher rates of tobacco use and poor lipid profiles. A previous systematic review by Maraka et al. reported low-quality but consistent evidence linking sex steroid therapy with dyslipidemia and increased risk of coronary artery disease among transgender individuals (28).

Myocardial infarction was found to have a pooled prevalence of 7% in our review, consistent with the findings of Alzahrani et al., who reported an MI prevalence of 7.2% in transgender men and 7.8% in transgender women (10). MI is a major cause of morbidity and mortality in this population (23). Although hormone therapy may contribute to this risk, it is important to consider the multifactorial nature of MI development in transgender individuals. In addition to clinical and treatment-related factors, researchers have identified strong associations between chronic social stressors, such as discrimination, health inequities, socioeconomic instability, and substance abuse, and increased inflammation, autonomic dysfunction, and cardiovascular risk (29, 30).

Publication bias was assessed using a funnel plot analysis of all major cardiovascular outcomes. A symmetrical distribution of studies was observed for hypertension and stroke/CVA, suggesting a minimal risk of publication bias for these outcomes. However, asymmetry in the funnel plots for CAD and MI raises concerns regarding potential bias, likely due to the underreporting of negative or null findings. Despite these limitations, the overall strength of the evidence from the included observational studies adds substantial value to the existing body of knowledge.

In summary, this systematic review and meta-analysis offer important evidence highlighting the substantial burden of cardiovascular disease among transgender individuals. The elevated pooled prevalence rates of hypertension, stroke, CAD, and MI underscore the need for sex-affirming, accessible, and preventive cardiovascular care tailored to the needs of transgender populations. These findings support the integration of regular cardiovascular risk assessment into routine healthcare for transgender individuals, especially those undergoing hormone therapy. There is a critical need for future large-scale, prospective cohort studies to better delineate the cardiovascular risk profile in transgender individuals stratified by gender identity, hormone use, and duration to guide clinical practice and policymaking more effectively.

### Strengths and limitations

This systematic review is among the few to focus exclusively on the prevalence of cardiovascular diseases in transgender individuals, providing pooled estimates of hypertension, myocardial infarction, stroke, and coronary artery disease. The study adhered to the PRISMA guidelines, used well-defined inclusion criteria, and applied the JBI critical appraisal tool to assess the study quality, enhancing the reliability and public health relevance of the findings. However, this study had several limitations. The search was limited to selected databases and English-language publications, which may have introduced language bias and resulted in exclusion of potentially relevant studies published in other languages. Only observational studies were included, as randomised controlled trials evaluating cardiovascular outcomes of gender-affirming hormone therapy are currently lacking and were outside the scope of this review. Most included studies lacked detailed data on hormone therapy exposure, limiting assessment of its potential association with cardiovascular outcomes. Cardiovascular risk among transgender individuals is likely multifactorial, involving both biological determinants, such as hormone-related metabolic and vascular effects, and social determinants including stigma, discrimination, healthcare barriers, socioeconomic instability, and chronic psychosocial stress. Additionally, cisgender individuals were not included in the comparison group, which limits the contextual interpretation of cardiovascular risk burden. The generalizability of these findings is limited by the predominance of studies from the United States and South Asia, variable healthcare access, reliance on self-reported diagnoses in several datasets, and underrepresentation of older transgender adults. These factors may influence prevalence estimates and limit extrapolation to regions with universal healthcare or structured gender-affirming care pathways. Substantial heterogeneity and possible publication bias were observed across several outcomes, likely reflecting variations in study design, participant characteristics, geographic settings, healthcare access, and hormone therapy exposure. Although subgroup analyses were considered, the limited number of studies and inconsistent reporting of covariates precluded robust subgroup or meta-regression analyses. Additionally, some pooled estimates were disproportionately influenced by large population-based datasets, which may have affected weighting and overall pooled prevalence estimates. Funnel plot interpretation may also be unreliable for outcomes with fewer than 10 included studies, limiting the robustness of publication bias assessment. Therefore, pooled prevalence estimates should be interpreted cautiously and viewed as indicative rather than definitive estimates of cardiovascular disease burden among transgender populations. Despite these limitations, this review provides essential evidence on cardiovascular health in transgender populations and highlights the need for inclusive cardiovascular screening and targeted care strategies.

## Conclusion and recommendations

This systematic review and meta-analysis highlight the significant burden of cardiovascular diseases, including hypertension, myocardial infarction, stroke, and coronary artery disease, among transgender individuals. These findings underscore the urgent need for greater clinical attention to cardiovascular risk assessments in this population, which remains underserved and medically vulnerable. The consistently observed prevalence of cardiovascular morbidities across heterogeneous settings suggests that transgender individuals represent a population with a meaningful cardiovascular disease burden, warranting inclusive application of existing cardiovascular screening guidelines rather than novel or intensified surveillance strategies. However, these findings should be interpreted cautiously given the limited number of included studies, substantial heterogeneity, and methodological variability across the available evidence.

### Recommendations

- Routine cardiovascular screening should be integrated into transgender healthcare, especially in individuals undergoing hormone therapy.
- Future studies should include detailed data on hormone use, duration, and type to better understand their association with cardiovascular outcomes.
- Comparative studies involving cisgender populations are needed to contextualise cardiovascular risk in transgender individuals.
- Health policies must be inclusive and promote accessible and non-discriminatory healthcare services tailored to the unique needs of transgender populations.

## Data Availability

The original contributions presented in the study are included in the article/supplementary material, further inquiries can be directed to the corresponding authors.
